# Cancer-associated fibroblast cell surface markers as potential biomarkers or therapeutic targets in lung cancer

**DOI:** 10.20517/cdr.2024.55

**Published:** 2024-09-10

**Authors:** Samaneh Tokhanbigli, Mehra Haghi, Kamal Dua, Brian Gregory George Oliver

**Affiliations:** ^1^School of Life Science, University of Technology Sydney, Sydney, NSW 2007, Australia.; ^2^Respiratory and Cellular Molecular Biology Group, Woolcock Institute of Medical Research, Sydney, NSW 2037, Australia.; ^3^Discipline of Pharmacy, Graduate School of Health, University of Technology Sydney, Sydney, NSW 2008, Australia.; ^4^Faculty of Health, Australian Research Centre in Complementary and Integrative Medicine, University of Technology Sydney, Sydney, NSW 2008, Australia.

**Keywords:** Fibroblasts, lung cancer, inflammation, targeted therapy, therapeutic markers

## Abstract

Cancer-associated fibroblasts (CAFs) are the vital constituent of the tumor microenvironment, and in communication with other cells, they contribute to tumor progression and metastasis. Fibroblasts are the proposed origin of CAFs, which are mediated by pro-inflammatory cytokines and the recruitment of immune cells akin to wound healing. Although various studies have identified different subpopulations of CAFs in lung cancer, the heterogeneity of CAFs, particularly in lung cancer, and their potential as a therapeutic target remain largely unknown. Notwithstanding CAFs were previously thought to have predominantly tumor-promoting features, their pro- or anti-tumorigenic properties may depend on various conditions and cell origins. The absence of distinct markers to identify CAF subpopulations presents obstacles to the successful therapeutic targeting and treatment of CAFs in cancer. Human clinical and animal studies targeting CAFs have shown that targeting CAFs exacerbates the disease progression, suggesting that subpopulations of CAFs may exert opposing functions in cancer progression. Therefore, it is essential to pinpoint specific markers capable of characterizing these subpopulations and revealing their mechanisms of function. The cell-specific surface markers of CAFs will serve as an initial step in investigating precise CAF subpopulations and their role in diagnosing and targeting therapy against cancer-promoting CAF subsets in lung cancer.

## INTRODUCTION

Global Cancer Statistics 2020 (GLOBOCAN) highlighted lung cancer as the second most commonly diagnosed, trailing only behind breast cancer, and yet the leading cause of death worldwide^[1]^. The etiology of lung cancer is multifaceted, implicating genetic and non-genetic factors. Genetic mutations play a central role in identifying several key driver mutations. Epigenetic modifications are other contributing factors to lung cancer^[2]^. Notably, smoking stands out as the main risk factor for all lung cancer^[3]^. The heterogenicity of the cells in the tumor microenvironment predominantly manifests during disease onset and progression. Therefore, this heterogenicity is the main obstacle in the way of innovative treatment resistance, such as immune therapy and targeted therapy^[4]^.

Different cellular, cytokine, and immune components regulate the tumor microenvironment, and cancer-associated fibroblasts (CAFs) play a seemingly ever‐increasing role in this context.

The tumor microenvironment and its components, such as fibroblasts, were first hinted at in the late 19th century, and after that, the tumor stromal component and its function were explored. Researchers discovered that the stromal cells, mainly fibroblasts, play a role in cancer cell support in the tumor microenvironment and orchestrate the broad range of activity in that environment^[5]^. At this point, these cells started gaining more research attention and were named CAFs.

Like other cancers, in lung cancer, CAFs constitute the major component of the stroma. In the lung, the normal stroma helps regulate the homeostasis and integrity of epithelial cells. In this regard, any secreted mediator or immune cells could alter the directional crosstalk between the stroma and epithelial cells^[6]^.

**Figure 1:**
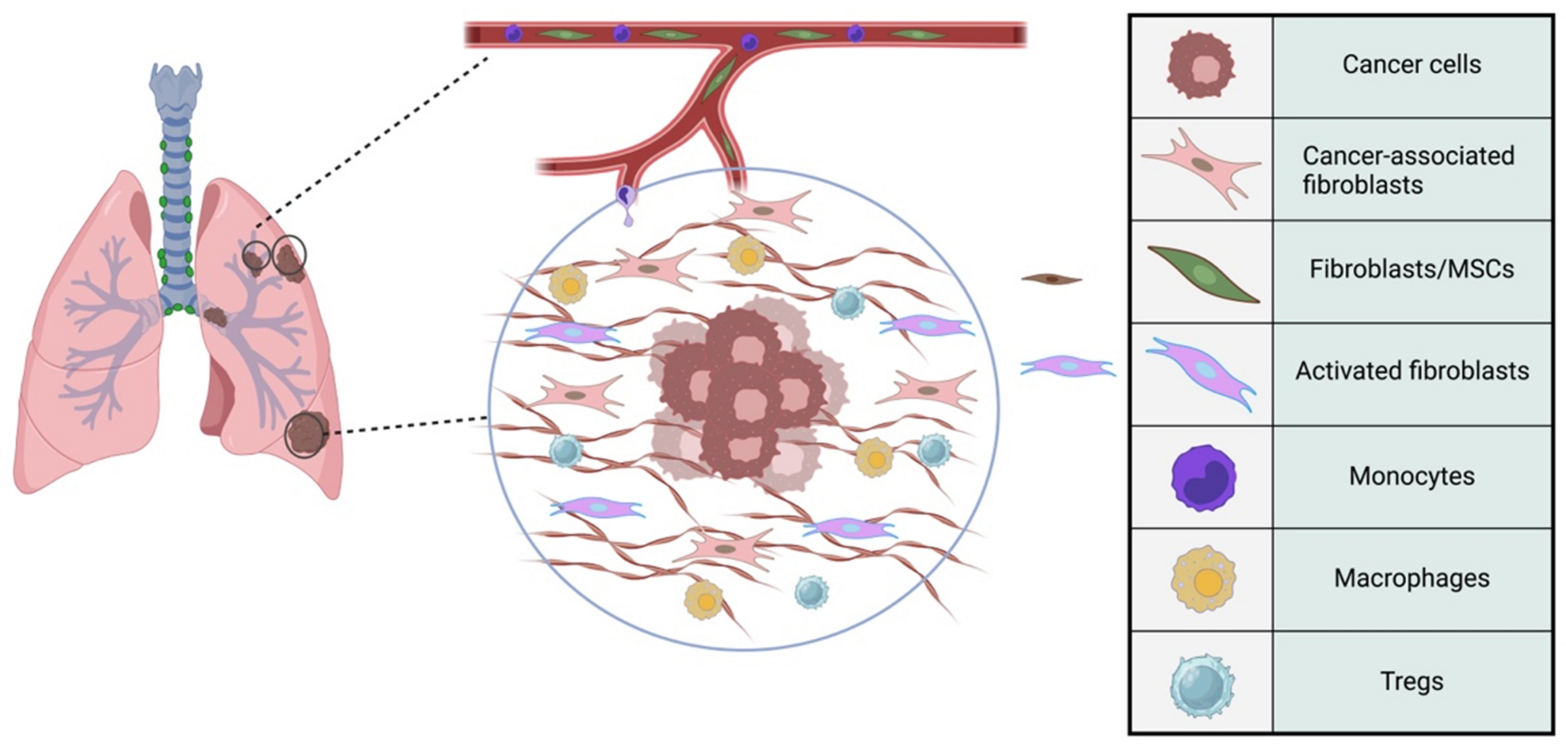
Schematic Crosstalk of CAFs and immune cells in the tumor microenvironment of lung cancer. CAFs orchestrate the tumor microenvironment, interacting with various cell types within the tumor microenvironment of lung cancer. An example of this immune component is cancer cells, MSCs, activated fibroblasts, monocytes, macrophages, and Tregs. MSCs could differentiate into CAFs and other cell types, supporting the tumor. Activated fibroblasts are involved in remodeling the ECM and promoting tumor invasion. Monocytes can differentiate into macrophages within the tumor microenvironment. Macrophages can have pro-tumorigenic roles, promoting tumor growth and suppressing the immune response. Tregs contribute to the immunosuppressive environment within the tumor, aiding in tumor evasion from the immune system. CAFs: Cancer-associated fibroblasts; MSCs: mesenchymal stem cells, Tregs: regulatory T cells; ECM: extracellular matrix.

The resting fibroblasts within the normal stroma get activated, and they differentiate into specialized cells, a process facilitated by releasing inflammatory mediators and recruiting immune cells akin to wound healing^[7]^. Through communication with tumor cells, CAFs contribute to many key tumor characteristics. They contribute to extracellular matrix (ECM) degradation via different pathways [e.g., matrix metalloproteinase (MMP) secretion] to facilitate tumor dissemination. At the same time, they orchestrate new ECM production to maintain the tumor structure^[8]^. Moreover, CAFs interact with immunosuppressive cells such as M2 macrophages, myeloid-derived suppressor cells (MDSCs), and regulatory T (Treg) cells, which further promote tumor progression and metastasis^[8]^ [Figure 1].

In addition to resting fibroblasts in tumor stroma, various origins have been suggested for CAFs in lung cancer, including tissue fibroblasts, bone marrow progenitor cells, pericytes, and epithelial cells via epithelial-mesenchymal transition (EMT)^[9-12]^.

While the tumor microenvironment can prompt the transformation of resident fibroblasts into CAFs^[13]^, this process is not unidirectional and can be reversed^[14]^.

In addition to the proposed origins of CAFs in lung cancer, a recent study has identified the contribution of tumor-associated macrophages (TAMs) to the formation of CAFs in non-small cell lung cancer (NSCLC). By single-cell RNA sequencing (scRNA-seq), they identified macrophage-to-myofibroblast transition (MMT) as a mechanism by which M2 macrophages give rise to CAFs in NSCLC through a Smad3-centric gene network in experimental models and humans^[15]^. However, CAFs' origins might be different for different cancers.

CAFs function in tumor progression through inducing drug resistance and angiogenesis^[16]^. In lung cancer, the transition of fibroblasts into CAFs is mediated by the profibrotic cytokine transforming growth factor beta 1 (TGF-β1), produced from epithelial cells, much like other wound healing processes. A microarray study in lung cancer patients demonstrated that TGF-β1-related genes contributed to cell invasion, angiogenesis, and immune invasion^[17]^ and are highly upregulated compared to normal tissues^[18]^.

**Figure 2:**
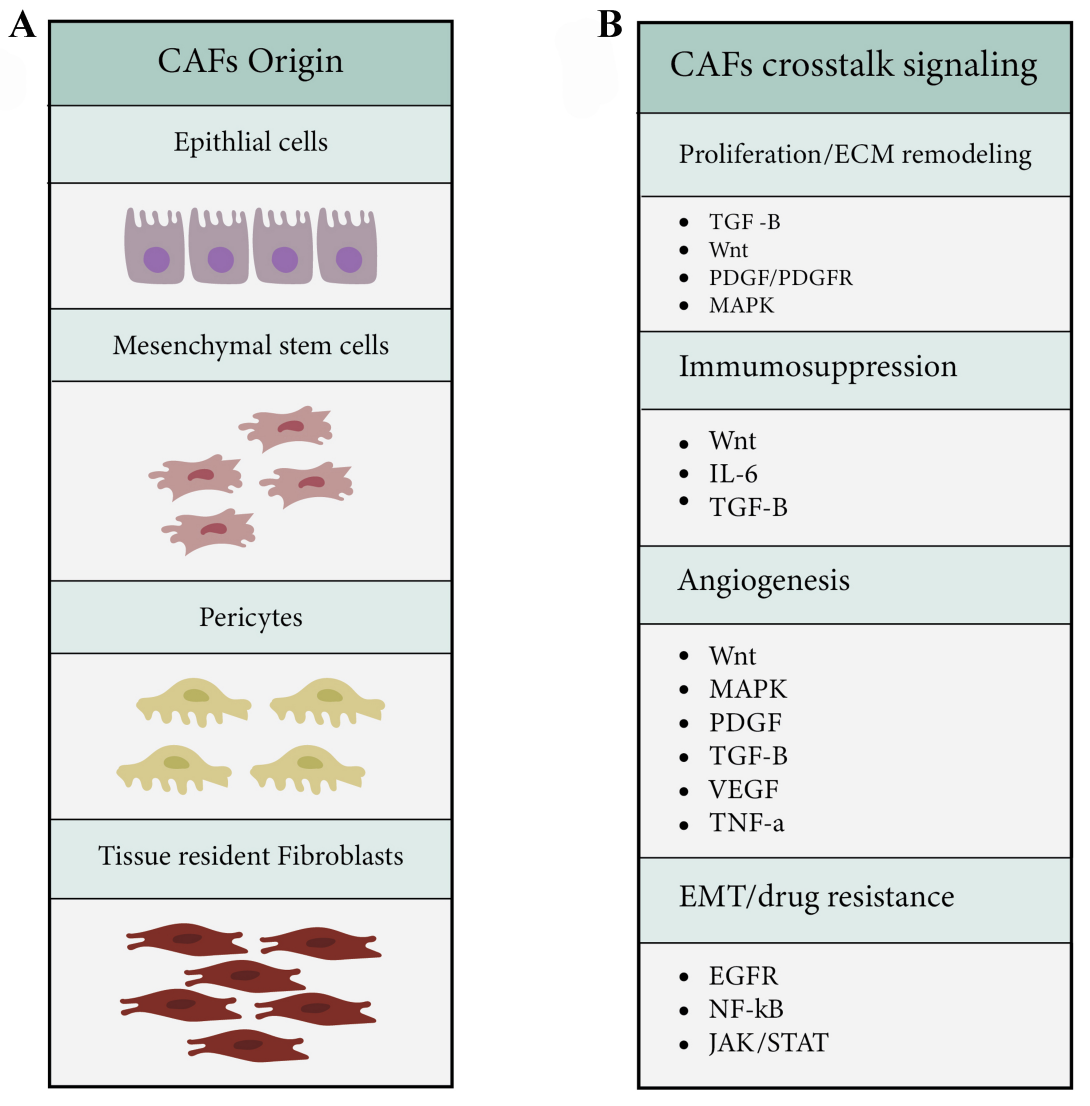
The origin of CAFs and their crosstalk signaling pathways regulating tumor microenvironment. (A) CAFs in lung cancer are potentially derived from tissue-resident fibroblasts, mesenchymal stem cells, epithelial cells, and pericytes; (B) These CAFs modulate the tumor microenvironment by activating a variety of signaling pathways, including TGF-β, Wnt/β-catenin, MAPK, IL6, EGFR, JAK/STAT, and NF-κB, resulting in the orchestration of processes such as proliferation and ECM remodeling, immunosuppression, EMT, drug resistance, and angiogenesis. CAFs: Cancer-associated fibroblasts; TGF-β: transforming growth factor beta; MAPK: mitogen-activated protein kinases; IL: interleukin; EGFR: epidermal growth factor receptor; JAK/STAT: Janus kinases/signal transducers and activators of transcription; NF-κB: nuclear factor kappa-light-chain-enhancer of activated B cells; ECM: extracellular matrix, EMT: epithelial-mesenchymal transition.

Another activated signaling pathway in lung cancer CAFs is the platelet-derived growth factor receptors (PDGF/PDGFR) signaling pathway and its downstream component, mitogen-activated protein kinase (MAPK). PDGFR regulates the proliferation of fibroblasts, modulates angiogenesis, enhances CAF‐mediated ECM remodeling, and promotes tumor invasion in lung adenocarcinoma cells^[19]^. Lung cancer CAFs produce inflammatory mediators such as interleukin (IL)-6, IL-8, IL-17, IL-22, tumor necrosis factor (TNF)-α, and vascular endothelial growth factor (VEGF) to support progression, invasion, and angiogenesis. The hypoxic environment of lung cancer provokes the expression of hypoxia-inducible factor 1-alpha (HIF-1α) in fibroblasts and facilitates their transformation to CAFs [Figure 2A and B]^[20,21]^.

As the number of CAFs increases, patients generally have a poorer prognosis and lower survival rates. Although the critical role of CAFs in the tumor microenvironment is now better understood, the heterogeneity of these populations, particularly in lung cancer, is still largely unknown.

There have been clinical cohorts and animal studies targeting CAFs that have exacerbated the disease progression^[22-24]^, implying that distinct subpopulations of CAFs may exert opposing roles in cancer progression.

It is widely held that CAFs are a cellular state rather than a specific cell type; however, the lack of specific cell markers that would allow therapeutic targeting makes it challenging to effectively target and treat CAFs in cancer.

This review aims to offer a detailed and thorough overview of the current comprehension of the potential biomarkers of CAFs presented in Table 1 in NSCLC and explore the potential implications of these findings for treating NSCLC. In addition, the specific markers recently identified through scRNA-seq in NSCLC will be discussed.

**Table 1:** List of potential CAF biomarkers in lung cancer

**CAF marker**	**Bio function in tumor microenvironment**	**Expressing cell**	**Ref.**
α-SMA	Promoting tumorigenesis, ECM remodeling, proliferation, drug resistance, immune tolerance through T and NK, DC cells exhaustion, Th2 responses, M2 and Treg recruitment	Vascular, muscular cells pericytes, fibroblasts	[25-27]
FAP	Promoting tumorigenesis, tumor invasion, ECM remodeling, immune tolerance, Th2 responses, M2 and Treg recruitment	Rarely expressed in normal tissues, epithelial tumor cells, M2 macrophages, tumor cells, tumor-promoting mesenchymal stromal cells, fibroblastic stromal cells	[27-30]
FSP1	ECM remodeling, tumor development, metastasis through VEGF-A and TNC-C, immune tolerance through M2 polarization recruitment	Fibroblasts, epithelial and endothelial cells	[31]
PDPN	tumor development, immune tolerance through T cell anergy, M2 polarization, and Treg recruitment	Endothelial cells	[32,33]
Collagen	ECM remodeling, angiogenesis, metastasis, immune suppression through M2 recruitment	Fibroblasts, tumor cells, endothelial cells	[34-36]
Vimentin	EMT mediated metastasis, tumorigenesis, and tumor development	Fibroblasts, mesenchymal cells	[37,38]
ITGA11	EMT, tumor progression	Mesenchymal cells	[39-41]
PDGFRα/β	Tumor development, immune evasion via T cell exhaustion and M2 polarization, angiogenesis	Fibroblasts, vascular smooth muscle cells, pericytes	[42,43]
CD10/GPR77	Inflammatory signaling, supporting CSC survival, promoting tumorigenesis, chemoresistance	Bone marrow mesenchymal stem cells, pre-B lymphocytes	[44,45]
TNC	ECM remodeling, EMT-mediated metastasis, angiogenesis and immunomodulation, tumor development	Fibroblasts, tumor cells, endothelial cells	[46-48]
Periostin	Tumor proliferation, metastasis	Fibroblasts, tumor cells, mesenchymal stem cells	[49,50]
Caveolin-1	ECM remodeling and desmoplastic reaction, cancer cell migration, and invasion	Adipocytes, endothelial cells, epithelial cells, pneumocytes, and fibroblasts	[51,52]

CAF: Cancer-associated fibroblast; α-SMA: alpha-smooth muscle actin; ECM: extracellular matrix; NK: natural killer; DC: dendritic cell; Th: T helper; M2: macrophage; Treg: T regulatory; FAP: fibroblast activating protein; FSP: fibroblast-specific protein; VEGF: vascular endothelial growth factor; TNC: tenascin; PDPN: podoplanin; EMT: epithelial-mesenchymal transition; ITGA11: α11β1 integrin; PDGFRα/β: platelet-derived growth factor receptor α/β; GPR: G-protein coupled receptor; CSC: cancer stem cell.

## ALPHA-SMOOTH MUSCLE ACTIN POSITIVE CAFS

Numerous efforts have been made to identify a reliable marker for CAFs in NSCLC. Alpha smooth muscle actin (α-SMA), a marker commonly expressed by activated fibroblasts and associated with TGF-β1 stimulation, has been extensively studied and considered a traditional CAF marker^[53,54]^. However, the expression of α-SMA is not exclusive to fibroblasts and can be influenced by the tumor microenvironment; regarding the identification of cell surface markers, it is worth mentioning that fibroblasts and airway smooth muscle (ASM) cells both express α-SMA, which precludes differentiation of these two cells by cell surface markers. ASM cells are proposed as proliferative and contractile cells expressing high levels of α-SMA in asthmatic patients and contribute to airway remodeling in asthma. The α-SMA positive ASM cells exhibit more proliferative and immunoreactive properties, increasing the notion that these cells are responsible for thickening the airway in asthma^[55]^. Furthermore, it is shown that in severe asthma, myofibroblasts contribute to airway thickening asthma by migrating and/or differentiating ASM-like cells^[55]^. Isolated fibroblasts from asthmatic patients after activation with TGF-β1 acquire ASM phenotype *in vitro*^[55]^.

In lung cancer and α-SMA expressing CAFs, the obtained tumor tissues from 78 patients of NSCLC in different stages (I-III) have indicated that α-SMA is defined only by stromal fibroblasts and not cancer cells in NSCLC. Moreover, the clinicopathological features of NSCLC cancer correlate with α-SMA high expression. Interestingly, a higher expression of α-SMA was observed in squamous cell carcinoma (SCC) and adenocarcinoma samples. On the other hand, TGF-β1/Smad signaling is regarded as one of the CAF activation pathways. Unlike esophageal SCC, no significant correlation was detected between the levels of TGF-β1 in cancer cells and the levels of αSMA in stromal fibroblasts. Patients with high TGF-β1-expressing stromal fibroblasts had poor overall survival, indicating a potentially significant prognostic value of TGF-β1 and α-SMA within stromal fibroblasts^[56]^. In agreement with these findings, Alcaraz *et al.* reported that the immunostaining of obtained NSCLC-diagnosed tissues indicated that high expression of α-SMA in CAFs is correlated with a higher risk of recurrence and death in NSCLC patients^[57]^. However, the diagnostic value of α-SMA as a marker of CAFs is still controversial^[58]^.

## FIBROBLAST ACTIVATION PROTEIN 1

Fibroblast activation protein (FAP) is another potential CAF marker in various cancers^[59,60]^. It is involved in wound healing and is expressed during inflammatory conditions such as the tumor microenvironment but rarely on normal cells. In the tumor stroma, FAP is expressed by mesenchymal stem cells. CAFs play a significant role in angiogenesis and metastasis, and FAP is expressed in about 90% of stromal fibroblasts in different cancers, including lung cancer^[61,62]^. A study of 122 NSCLC patient samples revealed that tumors enriched with CAFs are more susceptible to lymphatic metastasis and angiogenesis. In addition, CAF-enriched tumors exhibited high expression levels of FAP, which was linked to poorly differentiated tumors and metastasis in SCC. Similar results were also observed in FAP knockout animal models of lung and colon cancers^[63]^. However, conflicting results have been reported regarding the function of FAP. *In vitro* studies showed that overexpression of FAP in SCC cell lines correlated with pro-tumorigenic features through the phosphoinositide 3-kinases (PI3K)/protein kinase B (Akt) and sonic hedgehog (Shh) signaling pathways^[64]^. An analysis of 59 NSCLC patient specimens after tumor resection indicated that high expression of FAP is a negative indicator of poor survival^[65]^.

Conversely, a larger cohort of NSCLC patients in Kilvaer *et al.*’s study showed favorable results of high expression of FAP in SCC patients but not in ADC^[58]^. This inconsistency has also been reported in other malignancies^[66]^. These discrepant results may depend on the infiltration status of CD3^+^/CD8^+^ T-cells. In this regard, chimeric antigen receptor (CAR) T-cell therapy targeting tumor-promoting stromal cells shows potential for inhibiting tumor growth and enhancing endogenous CD8^+^ T-cell antitumor responses. The designed retroviral CAR construct specific for the mouse FAP selectively reduces FAPhi stromal cells and inhibits the growth of various murine tumors, namely lung cancer^[67]^. Studies have shown that increased survival of NSCLC patients with high FAP expression may be positively related to forkhead box protein 3 (FOXP3) cells, which are involved in the chemotaxis of immunosuppressive cells^[68]^. This finding was observed in animal models of breast and colon cancers that received an oral DNA vaccine targeting FAP, which resulted in an inhibitory effect on tumor growth and lung metastasis in a CD8^+^ T-cell-dependent manner^[69]^. Despite these findings, caution is necessary when considering the clinical implementation of therapeutic strategies aimed at targeting FAP.

## FIBROBLAST-SPECIFIC PROTEIN-1

Fibroblast-specific protein-1 (FSP1), also recognized as the metastasis-associated protein S100A4, is a commonly employed marker for CAFs. The upregulation of S100A4 has been linked to unfavorable clinical outcomes in several types of cancer, including lung cancer^[70-72]^. A meta-analysis by Zhang *et al.* established the clinicopathological significance of S100A4 overexpression in the advancement and metastasis of NSCLC patients^[73]^.

Activated fibroblasts isolated from primary tumors of NSCLC patients displayed elevated expression levels of α-SMA, FAP, and S100A4 compared to normal fibroblasts^[74]^. Consistent with these findings, inhibiting S100A4 with small hairpin RNA (shRNA) or Niclosamide impeded nuclear factor kappa-light-chain-enhancer of activated B cells (NF-κB) activity and, subsequently, the expression of MMP9 in various lung cancer cell lines, ultimately curtailing their invasiveness^[72]^. Additionally, the suppression of S100A4 sensitized A549 cells to radiation therapy^[75]^. Hou *et al.* demonstrated that overexpression of S100A4 in lung cancer cells promoted cell proliferation and tumor development via the Wnt/β-catenin pathway by inhibiting starvation-induced autophagy^[76]^.

## PODOPLANIN

Podoplanin (PDPN) is a commonly used CAF marker implicated in the invasiveness of lung cancer cells^[77]^.

PDPN expression in poorly differentiated carcinomas of SCC has been observed, indicating its role in tumor invasion and lymph node metastasis^[78]^. An *in vivo* experiment was conducted in a lung cancer animal model by co-injecting cancer cells with two types of human fibroblasts (vascular adventitial or lung tissue-derived fibroblasts) transfected with a vector expressing PDPN. The study’s findings showed that the lung tumor formation was increased with overexpression of PDPN and was independent of the number of injected cancer cells. However, these models also demonstrated lymph node metastasis and a high risk of disease recurrence^[79]^.

PDPN is expressed in CAFs, lymphatic endothelium, and inflammatory macrophages in the tumor microenvironment. This expression is crucial in tumor development, ECM remodeling, and immunosuppression in lung cancer^[80]^. Studies have shown that NSCLC patients (ADC and SCC) with PDPN-positive CAFs have a shorter overall survival^[81-84]^. Moreover, PDPN-positive fibroblasts recruited to the tumor microenvironment are also a poor prognostic marker for lung cancer patients. A meta-analysis conducted on tumor-infiltrating PDPN^+^ fibroblasts showed that the overall survival of lung cancer patients decreased significantly with an increase in the recruitment of PDPN-positive fibroblasts^[85]^.

Although PDPN-positive CAFs are an independent marker for recurrence and short survival^[23]^, the presence of CD204-positive TAMs along with PDPN overexpressing CAFs is another marker associated with a high risk of recurrence in lung cancer patients^[81]^.

CD204 is a scavenger receptor highly expressed in macrophages with immunosuppressive and pro-tumorigenic properties. In lung adenocarcinoma patients, PDPN-positive CAFs have been shown to induce resistance to gefitinib, an epidermal growth factor receptor/tyrosine kinase inhibitor (EGFR-TKI) used to treat advanced NSCLC^[86]^. This resistance may be mediated by the secretion of hepatocyte growth factor (HGF) by PDPN-positive CAFs, which activates the mesenchymal-epithelial transition (MET) signaling pathway and promotes cancer cell survival and growth. Targeting the PDPN/MET signaling axis may be a potential therapeutic strategy to overcome resistance to EGFR-TKIs in lung adenocarcinoma patients.

## INTEGRIN α11

The ECM deposition is a critical process in tumor progression and metastasis, and it is accelerated via the action of TGF-β through the transdifferentiation and activation of myofibroblasts^[87,88]^. These extracellular proteins, such as collagen and fibronectin, are among the differentially expressed genes in CAFs compared with normal fibroblasts.

One of the commonly overexpressed markers in stromal fibroblasts and resident mesenchymal cells in lung fibrosis and NSCLC is integrin α11 (ITGA11)^[18,89,90]^. ITGA11-expressing fibroblasts in NSCLC contribute to tumor progression and the EMT process, and its high expression in NSCLC patients correlates with poor prognosis. In a study using A549 cells cocultured with ITGA11-positive stromal fibroblasts, the cells had a greater potential for tumorigenicity and growth in a severe combined immunodeficient (SCID) mice model by regulating the expression of insulin-like growth factor 2 (IGF2) in the fibroblasts^[89]^. In line with these findings, the cell invasion and tumor growth of NSCLC tumors were hampered in α11-/- xenograft NSCLC models^[91]^, followed by diminished stiffness of tumor stroma and downregulation of focal adhesion kinase (FAK) and protein tyrosine kinase 2 (PTK2) activity. ITGA11 is expressed in various types of CAFs in different cancers, and its expression is regulated by TGF-β1 and its downstream signaling pathway Smad in fibroblasts^[41,92-94]^. Since ITGA11 is expressed only in lung fibroblasts and is involved in fibroblast differentiation and recognition of collagen, it serves as a specific marker for CAFs in NSCLC^[91,95-97]^.

## VIMENTIN

The process of EMT is closely associated with the development and progression of lung cancer, with more than half of cases diagnosed with metastasis^[98]^. During EMT, there is a decrease in E-cadherin expression and an increase in vimentin expression. In solid tumors, it has been observed that vimentin expression is associated with invasion and poor patient survival^[99-101]^. In a genetically engineered mouse model with co-mutations for KRAS/liver kinase B1 (LKB1), whole-body vimentin knockout resulted in early-stage lung tumor development with less invasive foci. Both the knockout and wild-type models had CAFs positive for vimentin. Inhibiting vimentin expression in surrounding CAFs in these models and 3D culture significantly reduced invasion. This indicates that invasiveness is more closely related to vimentin expression in CAFs than in tumor cells^[102]^. In support of this notion, CAFs have been shown to enhance lung tumor cells’ migration and invasion ability by regulating metastasis-related genes, including vimentin and E-cadherin^[103]^.

Nevertheless, it is essential to note that although vimentin is commonly expressed on fibroblasts of all types and used as an identifying marker, it is not a specific marker of CAFs.

## PDGFRS

The PDGF/PDGFR axis is well studied and is known as an important receptor tyrosine kinase (RTK) pathway in cancer development, metastasis, angiogenesis, and stromal cell functions^[104,105]^.

Generally, NSCLC tumors expressing PDGF and their corresponding receptors have a poorer prognosis^[106]^. Tejada *et al.* demonstrated that the PDGFR alpha signaling might contribute to the recruitment of stromal-activated fibroblasts and tumor growth in lung cancer carcinoma models. Moreover, tumor development is reduced by inhibiting the PDGFRα-mediated signaling in CAFs^[107]^. Consistent with the findings above, MEDI-575, a human-neutralizing monoclonal antibody explicitly targeting PDGFRα, has been shown to reduce tumor growth in NSCLC cancer models through modulation of stromal fibroblasts. These results further support the notion that the interplay between CAFs and tumor cells plays a crucial role in tumor development and progression^[108]^.

Imatinib mesylate is a well-known tyrosine kinase inhibitor prescribed for various cancers, including chronic myelogenous leukemia, acute lymphocytic leukemia, and gastrointestinal stromal tumors^[109]^. PDGFR α and β are predominantly expressed on stromal fibroblasts in lung cancer, while PDGFs are expressed on cancer cell lines. *In vitro* and *in vivo* administration of imatinib inhibits the phosphorylation of PDGFRβ, Akt1/2, and extracellular signal-regulated kinase (ERK)1/2 in stromal cells and the proliferation of fibroblasts^[110]^.

Dasatinib, another FDA-approved PDGFR inhibitor, has also been shown to affect CAFs in lung cancer *in vitro* at nanomolar concentrations compared to other counterparts, including imatinib, nilotinib, and sorafenib^[111]^.

Therefore, PDGFR inhibitors or newly designed small molecules targeting PDGFRs may be a promising strategy to enhance the efficacy of conventional therapies in lung cancer.

## CD200

CD200, also known as OX-2, is a cell surface glycoprotein expressed by a wide variety of cells, including tumor cells, endothelial fibroblasts, and immune cells^[112]^. CD200R, the responsive receptor to CD200, is primarily expressed in myeloid cells such as macrophages, neutrophils, and mast cells^[113]^. Furthermore, the crosstalk between cancer cells and immune cells suggests therapeutic targets in cancer therapy. This immunoregulatory axis functions in different conditions, such as lung injury.

As previously noted, CAFs that express PDPN in lung cancer patients have been found to be resistant to EGFR/TKI drugs, such as Gefitinib, and are associated with poor outcomes. Recently, CD200 has been proposed as a marker for CAFs in lung cancer, and it has been shown that high expression of CD200 in CAFs increases their sensitivity to EGFR/TKI inhibitors, such as Gefitinib, and induces cancer cell apoptosis. Furthermore, in patients with resected lung adenocarcinomas who have high expression of CD200, administration of Gefitinib has been associated with more prolonged progression-free survival^[114]^.

Research on CD200R has also shown that its expression in stromal cells is associated with poor prognosis and recruitment of tumor-suppressive immune cells. Although an increased number of the transcription factor FoxP3^+^ Tregs and programmed cell death protein (PD-1)^+^ cells were observed in CD200R-expressing stromal cells, the expression of CD200 in NSCLC increased the levels of cytotoxic T lymphocytes (CTL), natural killer (NK) cells, and T helper (Th)1 cytokine, IL-2 and interferon-gamma (IFNγ)^[115-117]^. Preclinical studies have demonstrated that activation of CD200R signaling inhibits Ras, ERK, P38, and Jun N-terminal kinase (JNK) signaling pathways. Additionally, blocking CD200R abolishes cell proliferation through MAPK and Akt suppression^[118]^. In line with these results, analysis of dissected tumor samples from NSCLC patients demonstrated that 75% of infiltrated T cells had high levels of CD200R expression on their surface. Furthermore, the expression levels of other immune checkpoints, such as PD-1 and cytotoxic T-lymphocyte–associated antigen 4 (CTLA-4), were elevated in these T cells, indicating immune suppression and T cell exhaustion^[119]^. These findings support the development of a new treatment strategy targeting CD200/CD200R expressing CAFs in NSCLC.

## CD10^+^ GPR77^+^

CD10 and G protein-coupled receptor 77 (GPR77) are two markers identified as pro-tumoral markers in CAFs, specifically in NSCLC and breast cancer. In NSCLC, the CD10^+^ GPR77^+^ CAF niche is characterized by the persistent secretion of IL-6 and IL-8 cytokines. The maintained activation of NF-κB signaling, as well as the subsequent phosphorylation and acetylation of p65, promote and contribute to the formation of CD10^+^ GPR77^+^ CAFs in NSCLC^[44]^. Furthermore, CD10^+^ GPR77^+^ CAFs provide a niche that supports cancer stem cell (CSC) survival, which promotes tumor progression and chemoresistance. Therefore, targeting this specific subset of CAFs could be a practical approach to cancer treatment.

## TENASCIN-C

The glycoprotein tenascin-C (TNC) is an ECM-related component highly expressed in tumor stroma and plays a comprehensive role in cancer^[120]^. Mounting evidence demonstrates that the tumor ECM actively promotes malignancy^[121]^. TNC is an anti-adhesive molecule that mainly functions through the inhibition of fibronectin^[122-124]^, which promotes cell invasion and EMT in cancer. TNC also has mitogenic activity and promotes the activation of Wnt and MAPK signaling pathways^[125]^. Angiogenesis and immunomodulation are other characteristics of TNC in cancer promotion and development. The high level of TNC expression is not limited to the stromal cells^[126,127]^; it is also detectable in the sera of NSCLC patients and shows a correlation with tumor size, the spread of cancer to lymph nodes, and the overall survival of patients^[128,129]^. TNC also suppresses T cell activity in lung cancer^[130,131]^. Since TNC is overexpressed in CAFs and rarely detectable in normal tissues, it could be a promising therapeutic target for lung cancer.

## CAVEOLIN-1

Caveolin, an essential component of caveolae, is a family of scaffolding proteins consisting of caveolin-1, caveolin-2, and caveolin-3. While caveolin-1 is expressed in various terminally differentiated cells, its role in different malignancies is controversial^[132]^. It has been proposed that caveolin-1 regulates the tyrosine kinase signal transduction molecules, including the EGFR, H-RAS, and the Src family. Gastric cancer patients who express caveolin-1 have been shown to have poor progression-free survival^[133]^, and its expression has been found to contribute to tumor progression and metastasis in lung adenocarcinoma cell lines, with a positive correlation with the tumor stage^[134]^. Additionally, caveolin has been shown to play a role in integrin-mediated ECM remodeling in CAFs. In solid predominant adenocarcinoma cases, the expression of caveolin-1 by CAFs was higher^[52]^. Furthermore, gemcitabine-based chemotherapy resistance has been associated with high expression of caveolin-1 in lung adenocarcinoma^[135,136]^. However, Bertino *et al.* demonstrated that high expression of stromal caveolin-1 improved drug response and survival rate in recipients of nab-paclitaxel in a phase II trial of NSCLC patients^[137]^.

## PERIOSTIN

Periostin belongs to a class of proteins known as matricellular proteins that are capable of binding to both the ECM and cell surface receptors. Specifically, periostin is recognized for its involvement in developing airways and repairing alveolar epithelial tissue^[138]^. Periostin induces cell proliferation via integrin binding and is essential in fibrotic diseases such as idiopathic pulmonary fibrosis (IPF)^[139]^. Periostin is not only expressed in CAFs in NSCLC, but the activated fibroblasts in IPF also express periostin. The inhibition of periostin in IPF resulted in suppression of fibrosis in bleomycin models, and the high expression of this molecule is correlated with poor prognosis of NSCLC patients^[140-142]^.

On the other hand, periostin in A549 cells contributes to their resistance to cisplatin. This resistance occurs through activating signal transducers and activators of transcription 3 (STAT3) and Akt pathways and increasing survivin expression^[143]^. In addition, IPF patients are at significant risk of developing lung cancer, and these patients illustrate a more aggressive form of the disease compared with non-IPF patients. Periostin in IPF activates the tumorigenesis process of NSCLC through major inflammatory pathways such as TGF-β and Wnt/β-catenin, acting as signals that facilitate the advancement of NSCLC^[144,145]^. These findings indicate that periostin could be a favorable target in IPF and NSCLC^[146]^. In this regard, nintedanib as a tyrosine kinase inhibitor has been approved for treating lung adenocarcinoma and IPF. It also shows potential in LC by attenuating the immunosuppressive tumor microenvironment and enhancing the infiltration of cytotoxic CD8^+^ T cells. This mechanism may augment the responses to immune checkpoint blockade in an animal model of NSCLC^[147-149]^.

Based on the CAF markers in NSCLC introduced above, clinical trials are currently underway to test the therapeutic efficacy of these markers in the context of NSCLC therapy [Table 2].

**Table 2:** Clinical trials targeting CAFs in lung cancer

**Trial name**	**Identifier**	**Description**	**Status**
A phase I/II study of RO6874281 (a FAP-targeted immunocytokine) in combination with atezolizumab in patients with advanced solid tumors	NCT03386721	Investigates the safety and efficacy of RO6874281 combined with atezolizumab in advanced solid tumors, including lung cancer, by targeting FAP-expressing CAFs	Active, not recruiting
A study of crenolanib in patients with advanced or metastatic solid tumors	NCT01243346	Assesses the safety, tolerability, and preliminary efficacy of crenolanib, a PDGFR inhibitor, in targeting PDGFR-β-expressing CAFs in advanced or metastatic solid tumors, including lung cancer	Active, not recruiting
Phase I study of simtuzumab in combination with nivolumab in advanced solid tumors	NCT02472977	Examines the combination of simtuzumab, an anti-LOXL2 antibody, with nivolumab in advanced solid tumors, including lung cancer, to target CAFs	Completed
Phase I/II study of galunisertib in combination with nivolumab in recurrent NSCLC	NCT02423343	Explores the combination of galunisertib, a TGF-β receptor inhibitor, with nivolumab in recurrent NSCLC, aiming to modulate CAF activity	Completed
A study of volociximab (M200) in combination with paclitaxel and carboplatin in subjects with NSCLC	NCT00313701	Investigates volociximab, an anti-α5β1 integrin antibody, combined with chemotherapy in NSCLC to target integrin-expressing CAFs	Completed
A study of vismodegib (GDC-0449) in combination with erlotinib in patients with advanced NSCLC	NCT01064622	Examines the combination of vismodegib, a hedgehog pathway inhibitor, with erlotinib in advanced NSCLC to inhibit hedgehog pathway-activated CAFs	Completed
A study of FAP-IL2v and anti-PD-1 in patients with advanced solid tumors	NCT03875079	Evaluates the combination of FAP-IL2v with anti-PD-1 immunotherapy in advanced solid tumors, including lung cancer, to target CAFs and enhance the antitumor immune response	Recruiting

CAFs: Cancer-associated fibroblasts; FAP: fibroblast activation protein; PDGFR: platelet-derived growth factor receptors; TGF-β: transforming growth factor beta; NSCLC: non-small cell lung cancer; FAP-IL2v: a FAP-targeted interleukin-2 variant; PD-1: programmed cell death protein.

## SCRNA-SEQ REVEALS HETEROGENEOUS SUBPOPULATIONS OF CAFS IN LUNG CANCER

Through scRNA-seq, researchers have identified several subpopulations of CAFs, namely myofibroblast-like CAFs (myCAFs), inflammatory CAFs (iCAFs), and antigen-presenting CAFs (ApCAFs). Previously, CAFs were thought to have predominantly tumor-promoting features, but their pro- or anti-tumorigenic properties may depend on their cellular origin.

As shown in Table 3, three different approaches have been taken to define lung cancer CAF subpopulations. Kim *et al.* utilized scRNA-seq and pseudotime trajectory analysis to reveal the heterogeneity of lung CAFs. The THY1 marker was used to separate the active CAF subpopulation from surgically resected human lung adenocarcinoma tissues, which was associated with cancer cell invasion, migration, and poor prognosis in lung adenocarcinoma patients. Four major functionally distinct CAF branches (lineages) were identified: immunosuppressive, neoantigen-presenting, myofibroblastic, and proliferative CAFs. The study also identified specific markers for each CAF branch and confirmed the invasiveness-promoting role of ubiquitin-conjugating enzyme E2 T (UBE2T) and karyopherin subunit alpha 2 (KPNA2) in neoantigen-presenting CAFs^[150]^. Hu *et al.*, to address a better understanding of CAFs in NSCLC, established patient-derived fibroblasts (PDF) from NSCLC biopsies with specific oncogenic alterations such as EGFR mutations or anaplastic large-cell lymphoma kinase (ALK) fusions. The authors identify three major functional subtypes of CAFs expressing HGF and fibroblast growth factor (FGF) that have different impacts on treatments using EGFR and ALK TKIs. A link was also found between a patient's clinical response and the functional classification of CAFs from their tumors, suggesting potential clinical value. The expression of high HGF and FGF7 in certain types of fibroblasts (type I and II) is influenced by TGF-β signaling and downstream transcription factors^[151]^.

**Table 3:** CAF subpopulations identified in lung cancer

**CAF Subtype**	**Gene expression pattern**	**Ref.**
Cluster 1	• COL10A1 • Epithelial-mesenchymal transition-related genes • Expression of a high range of ECM proteins and TGF-β-associated genes • Upregulation of genes regulated by HOXB2 and FOXO1 (e.g., COL1A1, COL3A1 and COL6A1)	[152]
Cluster 2	• COL4A1 • Highest expression of myofibroblast marker ACTA2 • High expression of myogenesis (for example, MEF2C, MYH11 or ITGA7 • NOTCH pathway-related genes • Angiogenesis-related genes • Pericytes subset expressing RGS5 (specific marker for pericytes) • Upregulation of MEF2C (myogenic transcription factor) and ELK3-regulated genes • Downregulation of FOXO1 and MSC (myogenic inhibitor) regulated genes	
Cluster 5	• Lower myogenesis and high mTOR signature expression	
Cluster 6 (non-malignant fibroblasts)	• High levels of elastin • Low levels of collagens type I, III, V and VIII • Lack of collagen type VI expression	
Cluster 7	• Lower myogenesis and high mTOR signature expression	
Subtype I	Function marker • HGF^High^, FGF^High/Low^, p-SMAD2^Low^ Molecular marker • ADAMTS8, MMP3, MMP1, DLL4, … Suggested therapy: MET-plus-FGFR pathway blockade	[151]
Subtype II	Function marker • HGF^Low^, FGF^High^, p-SMAD2^Low^ Molecular marker • WNT16, TNFSF4, KRT7, MALL, … Suggested therapy: FGFR pathway blockade	
Subtype III	Function marker • HGF^Low^, FGF^Low^, p-SMAD2^High^ Molecular marker • RPS4Y1, EIF1AY, RPL10P9, HOXB9, …	
Branch 1	Immunosuppressive CAFs • IGFBP6, IFITM3, LGALS3 Apoptosis, immune system, cytokine signal	[150]
Branch 2	Antigen processing and presentation CAFs • UBE2T, TK1, CXCL12, KPNA2, and HMGB3 Cellular response to cytokine stimulus, antigen processing and presentation, and T cell receptor signaling pathway	
Branch 4	Myofibroblastic CAFs • Enriched markers of extracellular organization ECM, cytokine secretion such as CCL2 and TGF-β, cell migration	
Branch 5	Proliferative CAFs (the main identified population) • PRC1, AURKA Mitotic cell cycle process, cell division, cellular metabolic process	

CAF: Cancer-associated fibroblast; COL10A1: collagen type X alpha 1 chain; ECM: extracellular matrix; TGF-β: transforming growth factor beta; HOXB: homeobox B; FOXO1: forkhead box protein O1; COL1A1: collagen type I alpha 1 chain; COL3A1: collagen type III alpha 1 chain; COL6A1: collagen type VI alpha 1 chain; COL4A1: collagen type IV alpha 1 chain; ACTA2: actin alpha 2, smooth muscle; MEF2C: myocyte enhancer factor 2C; MYH11: myosin heavy chain 11; ITGA7: integrin subunit alpha 7; NOTCH: notch receptor 1; RGS5: regulator of G protein signaling 5; ELK3: ETS transcription factor ELK3; MSC: mTOR: mammalian target of rapamycin; HGF: hepatocyte growth factor; FGF: fibroblast growth factors; p-SMAD2: phosphorylated Sma- and Mad-related protein 2; ADAMTS8: a disintegrin and metalloproteinase with thrombospondin motifs 8; MMP: matrix metallopeptidase; DLL4: delta-like canonical Notch ligand 4; MET: mesenchymal–epithelial transformation; FGFR: fibroblast growth factor receptor; WNT16: Wnt family member 16; TNFSF4: TNF superfamily member 4; KRT7: keratin 7; MALL: mal, T cell differentiation protein-like; RPS4Y1: ribosomal protein S4 Y-linked 1; EIF1AY: eukaryotic translation initiation factor 1A Y-linked; RPL10P9: ribosomal protein L10; IGFBP6: insulin-like growth factor binding protein 6; IFITM3: interferon-induced transmembrane protein 3; LGALS3: galectin 3; UBE2T: ubiquitin-conjugating enzyme E2 T; TK1: thymidine kinase 1; CXCL: chemokine (C-X-C motif) ligand; KPNA2: karyopherin subunit alpha 2; HMGB3: high mobility group box 3; CCL: chemokine (CC motif) ligand; PRC1: protein regulator of cytokinesis 1; AURKA: Aurora kinase A.

**Figure 3:**
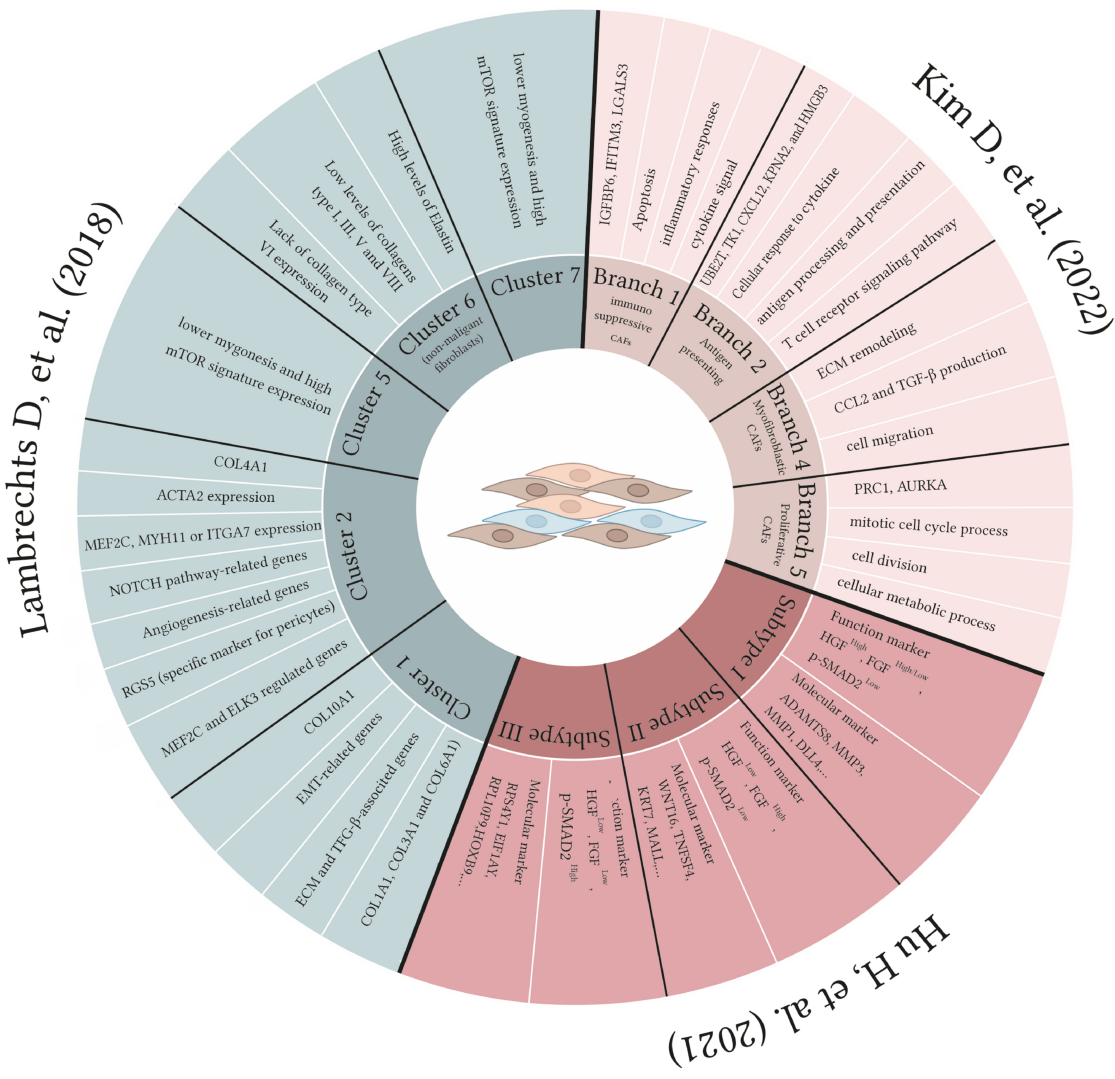
A schematic presentation of different branches/subtypes of CAFs in lung cancer CAFs. Single-cell RNAseq analysis revealed heterogeneity of CAFs in NSCLC. Kim *et al.* identified different branches of CAFs^[150]^, including immunosuppressive, ApCAFs, myCAFs, and proliferative CAFs. Hu *et al.* introduced three subtypes with various HGF and FGF expression levels^[151]^. Five unique CAFs were found in Lambrechts’s investigation; one was categorized as non-malignant fibroblasts^[152]^. CAFs: Cancer-associated fibroblasts; NSCLC: non-small cell lung cancer; ApCAFs: antigen-presenting CAFs; myCAFs: myofibroblast-like CAFs; HGF: hepatocyte growth factor; FGF: fibroblast growth factor.

The single-cell analysis of human lung tumors in Lambrechts *et al.*’s study revealed 52 stromal subtypes, including CAFs, compared to non-malignant samples. Five different types of fibroblasts were found, and non-cancerous samples were more abundant in cluster 6. The fibroblast subtypes were more enriched with ECM and distinct collagen components^[152]^. The component of each cluster is presented in Table 3 and Figure 3.

## ROLE OF CAFS IN DRUG RESISTANCE

Regarding the domination of CAFs in tumor microenvironments, especially in solid tumors, drug resistance could be the attributed feature of these cells. Therefore, it is crucial to identify the specifically expressed markers on these cells for targeted therapy to overcome drug resistance in NSCLC patients. Drug resistance could be provoked by supporting the survival of cancer cells and CSCs, tumor metabolism, and ECM regulation. In this regard, CD10^+^ GPR77^+^ CAFs, by supporting the formation of stem cell niches in lung cancer patients, help maintain the stemness of tumors and chemotherapeutic resistance in these patients. Su *et al.*’s study indicated that neutralizing monoclonal antibodies against GPR77 could reverse these adverse features in NSCLC patients^[44]^.

Zhao *et al.* demonstrated that oxidative stress induces TGF-β signaling in CAFs and boosts the synthesis of glycolytic byproducts, including L-lactate, pyruvate, and ketone bodies. These metabolites promote remodeling of the tumor microenvironment and cancer cell stemness and increase treatment resistance^[153]^.

Additionally, it has been shown that CAF-derived midkine as a heparin-binding growth factor is elevated in patients with ovarian, lung, and oral squamous cell carcinoma (OSCC) tissue. Secretion of the midkine from CAFs promotes cancer progression and cisplatin resistance via the elevated expression of lncRNA ANRIL^[154]^.

However, as we discussed before, not all the CAFs have the tumor suppressor feature and targeting them may exacerbate the patient’s condition. The major drug resistance mechanisms modulated by CAF in various cancers and affected pathways are presented in Table 4.

**Table 4:** CAF modulates cancer progression and drug resistance in different cancers

**CAF-mediated cancer progression**	**Secreted cytokines and chemokines from CAFs**	**Modulated pathways and signaling in cancer cells**	**Functions**	**Resistance to**	**Cancer types**	**Ref.**
Cancer cell survival	IL-6 CCL5 midkine SDF-1 IGF-1 HGF IL-8 CXCL12 CCL1	STAT3/PI3K/Akt signaling NF-κB pathway JAK2/STAT3 pathway TGF- signaling CXCR4/Wnt/-catenin signaling TGF-β/NF-κB pathway	Proliferation Chemoresistance EMT Cancer cell viability Cell survival	Cisplatin TKI Paclitaxel 5-fluorouracil Paclitaxel Gemcitabine	Ovarian cancer OSCC Lung cancer Breast cancer Pancreatic cancer Colorectal cancer Gastric cancer	[155-163]
CSC modulation	IL-17A TGF-β2 IL-6 IL-8 CD10 and GPR77	Wnt/β-catenin HGF/Met signaling Hh signaling	Self-renewal	Tamoxifen 5-fluorouracil Gefitinib Cetuximab	Breast cancer Colon cancer Melanoma Lung cancer	[44,164-166]
Cancer metabolism	HGF TIGAR	PI3K/Akt GPER/cAMP/PKA/CREB NF-κB NF-κB signaling	Cell survival Tumor progression	TKI Mitoxantrone Paclitaxel Topotecan Tamoxifen	NSCLC	[167-173]
ECM modulation	MMPs Caveolin-1 PDPN	Integrinβ1/PI3K/Akt pathway PI3K/Akt pathways	Tumor progression Tumor invasion	Doxorubicin Tamoxifen TKI	NSCLC Breast cancer PDAC	[86,91,174-182]

CAF: Cancer-associated fibroblast; IL-6: interleukin-6; CCL5: chemokine (CC motif) ligand 5; SDF-1: stromal cell-derived factor 1; IGF-1: insulin-like growth factor-1; HGF: hepatocyte growth factor; IL-8: interleukin 8; CXCL12: chemokine (C-X-C motif) ligand 12; CCL1: chemokine (CC motif) ligand 1; STAT3: signal transducers and activators of transcription 3; PI3K: phosphoinositide 3-kinases; Akt: protein kinase B; NF-κB: nuclear factor kappa-light-chain-enhancer of activated B cells; JAK2: Janus kinase 2; TGF-β: transforming growth factor beta; CXCR4: CXC chemokine receptor type 4; EMT: epithelial-mesenchymal transition; TKI: tyrosine kinase inhibitors; OSCC: oral squamous cell carcinoma; GPR77: G-protein-coupled receptor 77; Hh: hedgehog; TIGAR: TP53-induced glycolysis regulatory phosphatase; GPER: G protein-coupled estrogen receptor; cAMP: cyclic adenosine monophosphate; PKA: protein kinase A; CREB: cAMP response element-binding protein; ECM: extracellular matrix; MMPs: matrix metalloproteinases; PDPN: podoplanin; NSCLC: non-small cell lung cancer; PDAC: pancreatic ductal adenocarcinoma.

## CONCLUSION

Lung cancer is a prevalent and lethal form of cancer, with a five-year survival rate of approximately 19%. CAFs have attracted attention in targeted therapy for cancer due to their involvement in cancer development, progression, and metastasis^[183]^. The tumor microenvironment contains various subpopulations of CAFs with either tumor-promoting or tumor-suppressing features. CAFs have been shown to contribute to cancer progression through numerous signaling pathways^[184,185]^.

CAFs’ biological behavior, encompassing their interaction with cancer cells, the immune system, and metastasis and invasion, has been extensively studied. CAFs play a pivotal role in advancing tumorigenesis through mechanisms such as cytokine secretion, ECM modification, and EMT reprogramming. Notably, the rigidity of the ECM acts as a protective barrier shielding tumor cells from chemotherapy^[186]^. This ECM stiffening is notably augmented by collagen and the expression of integrins α11β1 by CAFs in NSCLC. Consequently, there has been a growing interest in using tumor organoids derived from patient’s tumor tissue as “cancer surrogates” to mimic tumor characteristics^[187,188]^. Nevertheless, it is imperative to acknowledge that these approaches have certain limitations, particularly in their ability to replicate the full spectrum of functions and attributes associated with CAFs, notably the absence of immune cells.

There are also unresolved questions about the relationship between CAFs at metastatic and primary sites. To illustrate, the analysis of RNA sequencing data from fibroblasts obtained from patients supports this observation. Results demonstrate that fibroblasts derived from different liver metastases of the same patient express some common markers like FAP but vary in the expression of other markers^[151,189]^. Nonetheless, it is worth noting that FAP may be considered a dependable marker for CAFs in the context of lung cancer. This is due to its expression in 90% of epithelial cells, and the inhibition has been shown to reduce tumor growth and increase anticancer medication uptake in tumor tissue^[63,189]^.

These findings indicate that fibroblast variations are not solely determined by their location and genetic factors, emphasizing the significance of understanding these variations to comprehend fibroblasts’ functional heterogeneity. Age and tumor location are also influential variables for CAF marker expression^[190,191]^.

Although S100A4 and α-smooth muscle actin are conventionally recognized as markers for lung cancer CAFs, their expression levels exhibit considerable variation among CAFs sourced from different biopsies. Interestingly, collagen type 1 α2 and αSMA expression correlated strongly with patients’ age, while PDGFRA and S100A4 expression correlated with the biopsy site^[192-195]^.

Considering the similarities in CAFs across different cancer types, it is important to note that these markers’ expression levels and functional roles can vary depending on the type and stage of cancer^[196]^. Nevertheless, some of these markers were investigated as therapeutic targets in lung cancer in different clinical trials.

For instance, Dasatinib, which binds to PDGFR, has demonstrated a promising effect on CAFs in lung cancer^[197]^.

Additionally, FAP inhibitors including a FAP-targeted immunocytokine in combination with Atezolizumab (an Anti-PD-L1 agent) (NCT03386721), the combination of a FAP-targeted interleukin-2 variant (FAP-IL2v) with anti-PD-1 immunotherapy (NCT03875079), and PDGFR inhibitors (NCT01243346) targeting PDGFR-β are currently undergoing clinical evaluation.

Nonetheless, identifying biomarkers for CAFs remains the initial and most important endeavor in this context. Numerous studies employing comparable methodologies such as RNAseq have identified various subtypes of CAFs with distinct markers and are expected to provide greater insight into these concerns and the diverse CAF populations. Identifying these biomarkers as potential therapeutic targets offers hope for treating lung cancer and other lung diseases, such as IPF.

In conclusion, due to the molecular diversity inherent to CAFs, it is imperative to conduct further investigations to comprehensively unravel the molecular mechanisms and clinical significance of CAFs in NSCLC and related respiratory conditions to develop targeted therapeutic approaches.

## DECLARATIONS

### Copyright

© The Author(s) 2024.
